# A GAP‐GTPase‐GDP‐P_i_ Intermediate Crystal Structure Analyzed by DFT Shows GTP Hydrolysis Involves Serial Proton Transfers

**DOI:** 10.1002/chem.201901627

**Published:** 2019-05-27

**Authors:** Robert W. Molt, Erika Pellegrini, Yi Jin

**Affiliations:** ^1^ Cardiff Catalysis Institute School of Chemistry Cardiff University Cardiff CF10 3AT UK; ^2^ Department of Biochemistry & Molecular Biology Indiana University School of Medicine Indianapolis Indiana 46202 USA; ^3^ ENSCO, Inc. 4849 North Wickham Road Melbourne Florida 32940 USA; ^4^ 9 European Molecular Biology Laboratory 71 Avenue des Martyrs, CS 90181 38042 Grenoble, Cedex 9 France

**Keywords:** computed proton states, GTPase, NMR spectroscopy, phosphoryl transfer, RhoA/RhoGAP

## Abstract

Cell signaling by small G proteins uses an ON to OFF signal based on conformational changes following the hydrolysis of GTP to GDP and release of dihydrogen phosphate (P_i_). The catalytic mechanism of GTP hydrolysis by RhoA is strongly accelerated by a GAP protein and is now well defined, but timing of inorganic phosphate release and signal change remains unresolved. We have generated a quaternary complex for RhoA‐GAP‐GDP‐P_i_. Its 1.75 Å crystal structure shows geometry for ionic and hydrogen bond coordination of GDP and P_i_ in an intermediate state. It enables the selection of a QM core for DFT exploration of a 20 H‐bonded network. This identifies serial locations of the two mobile protons from the original nucleophilic water molecule, showing how they move in three rational steps to form a stable quaternary complex. It also suggests how two additional proton transfer steps can facilitate P_i_ release.

Small G proteins are binary switching devices with the oncogenic Ras superfamily being the best studied. GTP binding gives the ON conformation which changes to OFF on hydrolysis of GTP to GDP.^1^ Transition between these two states is slow because GTP hydrolysis and GDP dissociation from the native G protein are inefficient in the absence of an external effector. These intrinsic properties are upregulated to deliver rapid signaling in vivo by GTPase activating proteins (GAPs), accelerating GTP hydrolysis (10^4^‐fold), and guanine nucleotide exchange factors (GEFs) facilitating GDP/GTP exchange.^1^ Inorganic phosphate is released after GTP hydrolysis, linked to conformational changes in Switches‐I and II from ON to OFF. The key unresolved question is: What proton transfers are needed to complete hydrolysis and release of GTP?^2^

Crystallography has provided direct information for the binding of GDP, of stable GTP analogues, and of transition‐state (TS) mimics for GTP hydrolysis through complex formation between GDP and an MF_*x*_^−^‐H_2_O moiety (MgF_3_^−^ or AlF_4_^−^) in the active site of a RhoGAP‐RhoA complex, whereas ^19^F NMR has shown the solution stability of such TSA complexes.^3^ DFT computation based on the MgF_3_^−^ transition‐state analogue (TSA) structure has identified the TS for hydrolysis as an attack of water on PG (γ‐phosphorus) with its two hydrogen atoms coordinated to the carbonyls of Gln63 and Thr37 (Figure 1).^3^ In the absence of acid/base catalysis, these mobile protons must relocate spontaneously to stable positions post‐TS in the intermediate complex before slow release of dihydrogen phosphate (P_i_).^4^ The lack of a structure for a GAP‐GTPase complex with GDP and P_i_ in the complete catalytic site has obscured understanding the relationship between GTP hydrolysis and the molecular mechanism of conformational switching. Existing structures for GDP‐P_i_ with small G proteins lack the catalytically essential GAP with its key Arg′ residue,^5^ so they cannot reveal the conformation switch following GTP hydrolysis and preceding dissociation of GAP.2a, 2b Moreover, the H‐bonds and positive charge contributed by Arg′ are essential for accurate analysis of the protonation state of the intermediate complex.^3^ Such a GDP‐P_i_ intermediate complex is a significant yet controversial element in the mechanism of GTP hydrolysis, hitherto modeled only from a low resolution TSA structure.^6^ Thus there is an expanding opportunity for DFT in modeling the role of proton activities in enzyme catalysis, especially for phosphoryl transfer reactions.^7^

**Figure 1:**
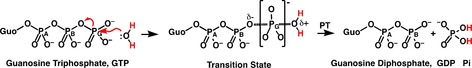
Hydrolysis of GTP to GDP and P_i_ via a transition state followed by proton transfer (PT).

NMR has not identified conformational changes in the course of rapid hydrolysis of GTP because of line broadening, fast dynamic interactions at the interface, and intermediate affinities for ternary complexes with GDP‐P_i_.^8^ FTIR has been used effectively to monitor GTP hydrolysis for a RasGAP‐Ras complex, though it has been unable to resolve the sequential order of consecutive fast events even at low temperature, while the shift imposed to match the simulated and experimental data makes it an indirect experimental method.^9^ Hence, the resolution of the timing of dissociation of P_i_ and GAP from the GTPase‐GDP complex relative to the ON–OFF conformational change of Switch‐I cries out for a GAP‐GTPase‐GDP‐P_i_ structure. Only this can show how inorganic phosphate is bound following (PB)O–PG bond cleavage, and also provide a structural platform for QM‐MM computation.

Well‐established techniques, such as soaking GTP into crystals of RhoGAP‐RhoA complex, do not work for direct acquisition of a RhoGAP‐RhoA‐GDP‐P_i_ intermediate complex because binding RhoGAP to RhoA is mediated by GTP at their interface.^10^ We also explored the formation of a RhoGAP‐RhoA‐GDP‐P_i_ complex in solution by NMR spectroscopy. ^13^C‐TROSY analysis of ^13^C,^15^N‐labeled RhoA‐GDP complexed with RhoGAP during stepwise addition of sodium phosphate was used to monitor phosphate binding (see the Supporting Information Experimental Section). The only chemical shift changes observed were correlated to amino acid residues on the protein surface, caused by a “salting‐out” effect, rather than to residues coordinating P_i_ in the active site. The data estimated a *K*_d_ for dissociation of P_i_ from RhoGAP‐RhoA‐GDP>1.0 m. This means that a co‐crystallization strategy is inappropriate (Figure S1, Supporting Information).

We therefore chose to use crystals preformed in a TS conformation and change their occupancy. We depleted high quality crystals of the RhoGAP‐RhoA‐GDP‐AlF_4_^−^ TSA complex^12^ of their bound AlF_4_^−^ using deferoxamine with minimal fluoride and magnesium. This initially gave crystals with occupancy of the active site by GDP‐MgF_3_^−^ (Figure S2, Supporting Information). Longer soaking in 200 mm P_i_ at low pH (pH 5.5) led to crystals of a RhoGAP‐RhoA‐GDP‐P_i_ complex diffracting to 1.78 Å (Figure 2, Table S1, Supporting Information). They showed electron density adjacent to GDP in a body‐centered tetrahedral assembly. It refined accurately for five atoms of a PO_4_ moiety bound in full occupancy (σ_A_‐weighted 2 *F*_o_−*F*_c_ countered at 1σ is 0.24 e Å^−3^, Figure S2, Supporting Information). We also obtained a 1.3 Å resolution X‐ray structure for a binary RhoA‐GDP product complex to support characterization of the post‐P_i_ release step, as the single extant structure (2.1 Å, 1FTN) lacks electron density for the key Switch‐II region (residues 61–78) (Table S1, Figure S3, Supporting Information).^13^

**Figure 2:**
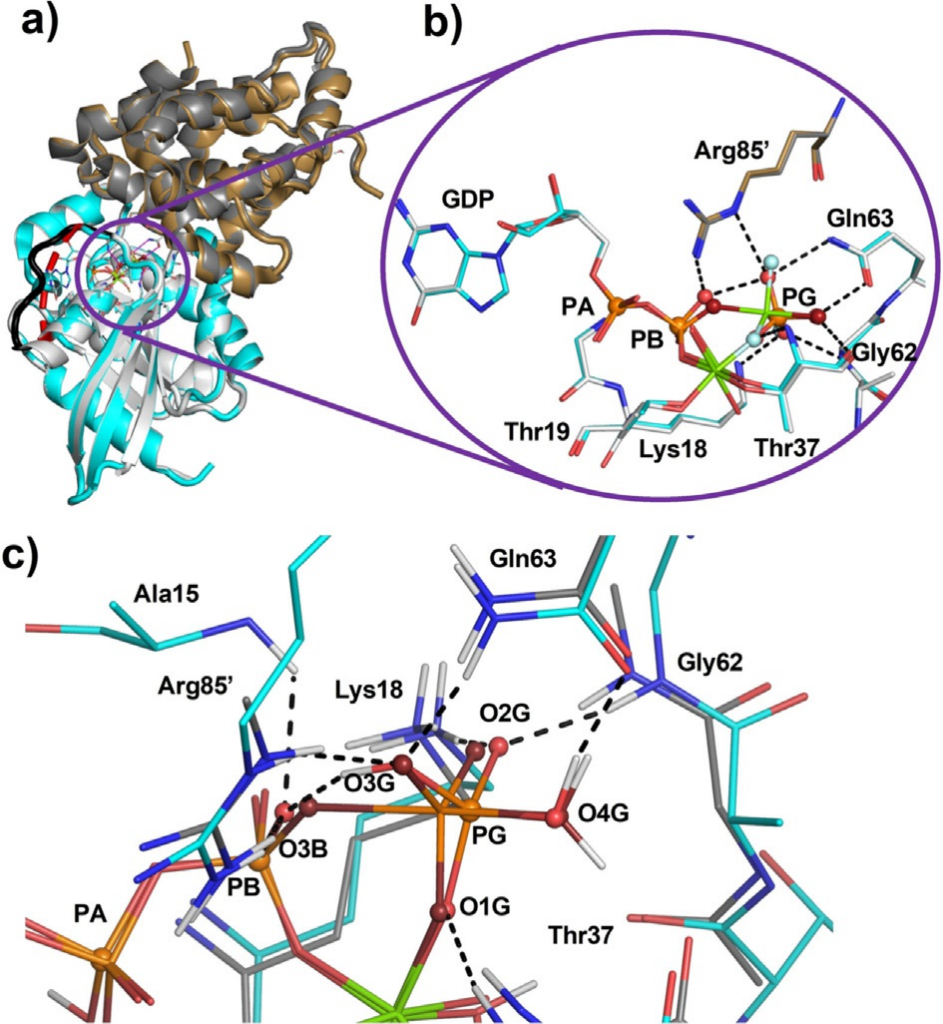
Structures of RhoGAP‐RhoA complexes. a) Tertiary structure alignment for RhoGAP‐RhoA‐GDP‐Pi (orange) and RhoA‐GAP‐GDP‐MgF_3_^‐^(gray)^[11]^ shows excellent fit except for residues 25–33 of Switch‐I. The ON conformation (green loop) is absent in the intermediate complex through disorder (broken red loop). b) Active site showing close correspondence of the GDP‐Pi intermediate (orange) aligned with MgF_3_^‐^(gray). c) Comparison of intermediate complex (cyan) with the computed TS (gray), both aligned on 1OW3, showing P−O bond rupture with 0.9 Å distance increase for O3B to PG, and very close alignment of oxygen atoms for phosphate intermediate (red spheres) and TS (O1G through O4G, ruby spheres). The 9 H‐bonds for the phosphate in the intermediate complex, to Ala15, Lys18, Thr37, Glu62, Gln63, and Arg85′ and O3B (LBHB) and its ligation to Mg^II^ are shown (black dashes; PA, PB and PG orange spheres).

The refined RhoGAP‐RhoA‐GDP‐P_i_ intermediate complex structure aligns well with that of the 1OW3 TSA complex with the notable exception of the disordered residues 25–33 of Switch‐I (Figure 2 a). Its tetrahedral PO_4_ moiety is closer (3.86 Å) than in Rab and Di‐Ras complexes lacking a GAP (4.0–4.2 Å) and as calculated for RasGAP‐Ras (4.1 Å).9e, 14 P_i_ is located by coordination to magnesium and Thr37_(N‐H)_, and by H‐bonds from Lys18, Gly62, and Arg85′ (Figure 2 b). The result shows very close proximity (2.54 Å) between O3G and O3B, characteristic of a low barrier H‐bond (LBHB), as observed in a Rab11‐GDP‐P_i_ complex (PDB ID: 1OIX).5d, 15 This organization places O4G at 2.63 Å from the Gln63 carbonyl oxygen atom but 3.08 Å from the Thr37 carbonyl oxygen atom, clearly indicating location of the proton on O4G oriented towards Gln63. Oxygen atoms O1G, O2G, and O4G of the phosphate map closely on their positions in the TSA structure (Figure 2 b). The Thr37 carbonyl oxygen atom and the guanidinium group of Arg85′ have the highest B factors (34.4 and 30.6 Å^2^) in the active site, significantly larger than for the other residues in this complex, suggesting their potential for movement in the P_i_ binding process. Overall, GDP aligns accurately with the nucleotide in 1OW3 (rmsd: 0.16 Å over 27 heavy atoms) showing the leaving oxygen atom O3B and PG have moved apart to increase their separation in the intermediate by 0.8 Å (Figure 2 c).

This intermediate has two significant features. Firstly, the Thr37 carbonyl oxygen atom is not H‐bonded to an O4G proton. Thus, it is poised to displace its own OH group as a stronger ligand for Mg^II^, as seen in RhoA‐GDP product structures (Figure S4, Supporting Information), and thence to initiate Switch‐I changing to the OFF conformation. Such an ROH ligand replacement by C=O fits the priority order X−O^−^>C=O> MeOH established for octahedral magnesium ligands.^16^ Secondly, because AlF_4_^−^ **⋅**2 H_2_O can dissociate out of the active site of the rigid RhoGAP‐RhoA‐GDP TSA complex crystal before P_i_ entry, along with the observed disorder of Switch‐I, it appears that phosphate dissociation can be enabled solely by a Switch‐I change from “ON” to “OFF”, with sufficient mobility demonstrated even in the solid state, without introducing an extra water into the active site (Figure S5 and S6, Supporting Information).

With complete structural information now available, we turned to DFT analysis to define full details of the H‐bond network, seeking the most favored protonation state for P_i_ in the intermediate complex and how it might be achieved through rational proton migrations. We also hoped it might inform on the nature of P_i_ release.2a, 2b, 9e The selected, manageable QM region includes the 18 amino acid moieties that contribute to the 20 H‐bonds framing the catalytic complex, the methyl triphosphate, and the nucleophilic water. Its 108 heavy atoms (210 total atoms; Figure 3) include six “methyl groups” at the boundary locked onto the coordinates of the parent carbon atoms in the RhoGAP‐RhoA‐GDP‐P_i_ structure.^17^ We used Kohn–Sham DFT (KS‐DFT) analysis for the computations with the M06‐2X functional formulation of KS‐DFT as described in earlier work (see the Supporting Information Experimental Section).^18^ Since O1G and O2G retain essentially the same binary coordination as in the intermediate structure, we focused on the protonation of O3G, O3B, and O4G variously orientated towards the three H‐bond acceptors of Gln63_(C=O)_, Thr37_(C=O)_, and O3B and explored a range of isomeric configurations (Figure 4 b–f, see the Supporting Information Methods Section). We found it useful to combine the H‐bond length (*r*_H–A_) and angle (∠ D‐H‐A) as the quotient Q_DA_ (∠ d‐H‐A**/** *r*_H–A_) giving an experimental device for gauging relative H‐bond contributions in the computed isomeric structures (Table S2, Supporting Information).

**Figure 3:**
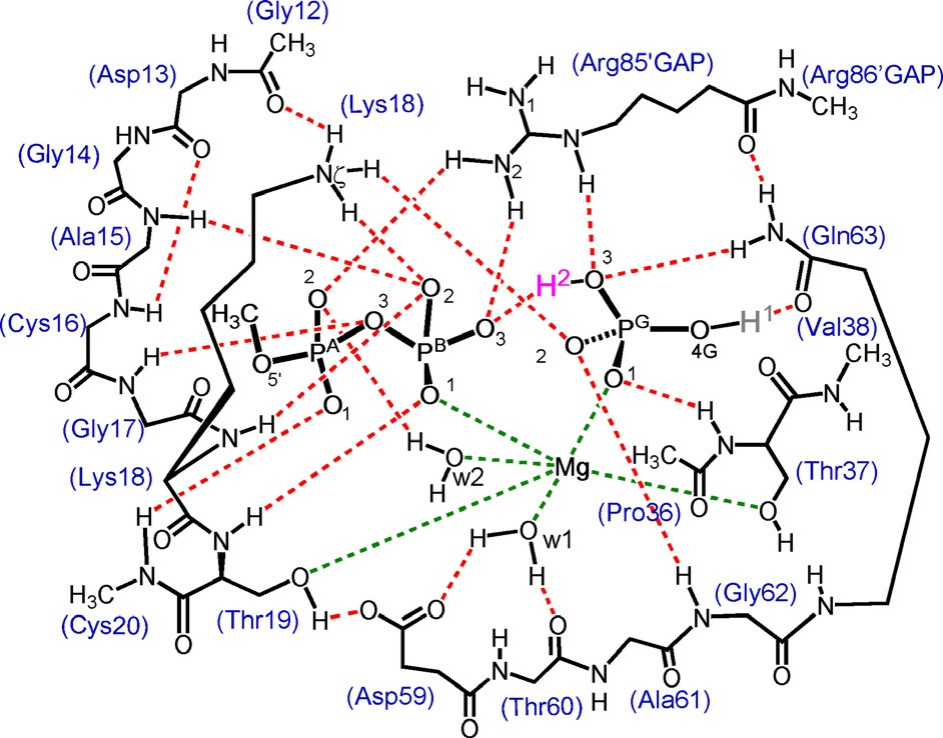
QM‐derived intermediate model for GDP‐Pi after GTP with 108 heavy atoms, with H‐bond network for the catalytic region (red dashes) with ligands coordinated to Mg (green dashes). Amino acid residues numbered according to the RhoA sequence plus Arg85′ from RhoGAP. A proton (red) is placed on O3G, consistent with the 2.5 Å separation of O3B and O3G (six CH3 groups are “locked” at the QM zone boundary). All the atoms are named using the IUPAC nomenclature.^19^.

**Figure 4:**
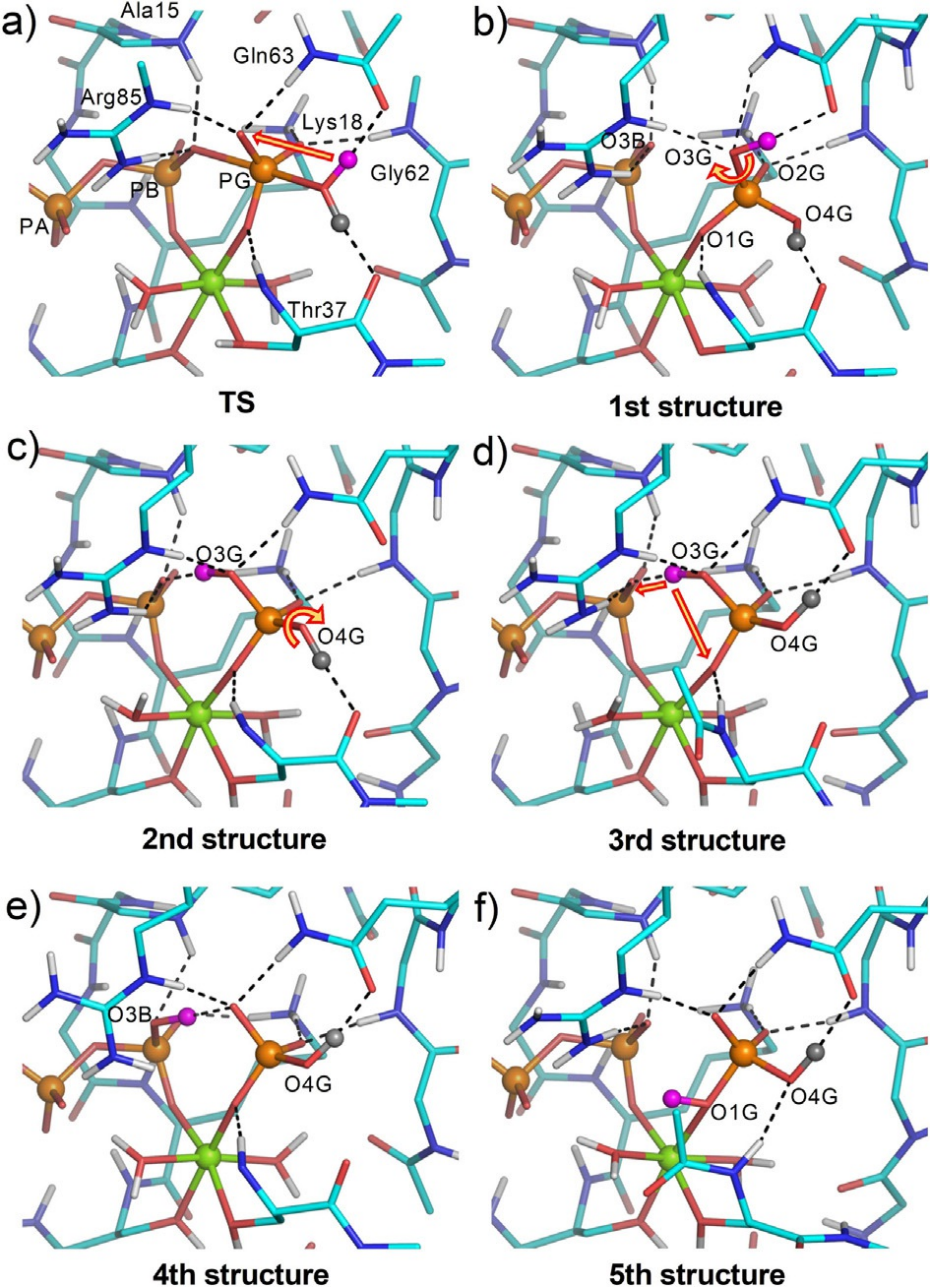
DFT computed structures for the active site of RhoGAP‐RhoA. a) TS structure has two Hs on O4G (H^1^ gray & H^2^ magenta) and 9 H‐bonds to Pi and O3B. b) Intermediate structure with H^1^ on O4G directed at Thr37 and H2 on O3G directed at Gln63. c) Rotating PG‐O3G bond places H^2^ in an LBHB with O3B while H^1^ coordinates Thr37 in a 9 H‐bond structure. d) Rotation of the PG‐O4G bond redirects H^1^ from Thr37 to Gln63 and retains the LBHB giving the most favored geometry. e) H^2^ moves from O3G to O3B resulting in a longer H‐bond to O3G and changed coordination for Arg85′. f) H^1^ is unchanged and H^2^ moves to O1G and makes no H‐bond (H‐bonds, black dashes; PA, PB and PG orange spheres; Mg^II^, green sphere).

We planned a succession of structures (Figure S7, Supporting Information) that starts from the TS computation.^3^ This has two mobile protons on O4G, the nucleophilic oxygen, 9 H‐bonds and a high mean Q_DA_ (96° Å^−1^) ((Figure 4 a and Table S2 entry 1, Supporting Information). The first computed structure retains the O4G proton H^1^ directed at Thr37_(C=O)_ as in the TS and H^2^ shifted to O3G and directed at Gln63_(C=O)_ (Figure 4 b). Its main features are an O3G to O3B distance of 3.3 Å, well outside Van der Waals separation, and no LBHB (Figure 4 b and Table S2, entry 2, Supporting Information). Mechanisms for this post TS proton transfer have been controversial, because there is no obvious catalyst for this change.^6^, ^20^ Our computed TS and 1st structure identify this transfer as involving electron donation from 3 oxygen atoms to H^2^ with uninterrupted H‐bonding to Gln63, linked to the major conversion of a tbp phosphoryl complex into a tetrahedral phosphate. This is beyond the scope of existing models yet seems eminently plausible.^20^ Very significantly, this first isomeric structure has an O_B_‐P_G_‐O_G_ angle of 176°, thus maintaining the “in‐line” character of the TS to the first intermediate complex, also manifest in the X‐ray structure (Figure 2 c). This angle changes to 150° only on subsequent formation of the LBHB between O3G and O3B (Figure 4 c), conflicting with the need for “bending the formed P−O bond for optimal phosphoryl transfer”, advocated elsewhere.^21^

The 2nd computed structure leaves H^1^ on O4G directed at Thr37_(C=O)_ with H^2^ on O3G now coordinating O3B, a change that requires simple rotation of the PG−O3B bond (Figure 4 c). It has 9 H‐bonds with the strongest being a LBHB to O3B (2.45 Å) and a satisfactory mean Q_DA_ 95° Å^−1^ (Figure S4c, Table S2, entry 3, Supporting Information). The 3rd structure has H^1^ on O4G reoriented to coordinate Gln63_(C=O)_ with H^2^ on O3G (Figure 4 d). This structure has 9 H‐bonds and maps closely on the second structure. A small improvement for its H‐bonds and a shorter LBHB (2.43 Å, angle 170°) results in a structural core with a mean Q_DA_ of 99° Å^−1^ (Figure S4d, Table S2, entry 4, Supporting Information), giving it the top rank of all six DFT structures analyzed (Figure 4 a–f). The 4th structure has H^1^ on O4G redirected at Gln63_(C=O)_ and H^2^ on O3B oriented at O3G (Figure 4 e). It has 7 H‐bonds overall, though the one from O3B to O3G is no longer a LBHB (2.57 Å), with a mean Q_DA_ 98° Å^−1^. Significantly, Thr37_(C=O)_ has moved 0.6 Å away from PG (Table S2, entry 5, Supporting Information).

The nonreversibility of P−O bond cleavage in the hydrolysis of GTP has been established by ^18^O isotope studies.2a Our result on LBHB for these two isomers in the 3rd and 4th structures now provides a clear explanation for this behavior: O3B cannot be a nucleophile towards PG: by accepting three H‐bonds from O3G, Arg85′, and Ala15 it has no electron pair available for in‐line nucleophilic interaction with PG. This is a powerful example of phosphoryl transfer being suppressed by H‐bonding between nucleophile and phosphoryl oxygen that denies bonding orbital overlap.^21^

The above results establish the most favored location for the migrating protons. H^1^ is located on O4G and coordination to Gln63 is better than to Thr37. H^2^ is preferentially bonded to O3G giving a shorter LBHB to O3B (2.43 Å) than for the alternate situation (2.57 Å) though the Q_DA_ scores are very close. This stable intermediate complex, implicit in the 6R3V crystal structure, is achieved in three sequential steps: H^2^ first shifts from O4G to O3G in the post‐TS separation of the γ‐phosphoryl group, which pivots 30° around a stationary O1G as its geometry changes from tbp to a tetrahedron. H^2^ stays well‐coordinated to Gln63_(C=O)_ from start to finish of this complex event. Next, PG−O3G bond rotation aligns H^2^ with the O3B to form a LBHB bridging two anionic oxygen atoms. Finally, H^1^ on O4G is redirected from Thr37_(C=O)_ to Gln63_(C=O)_ (Figure 4 b–d). This three‐step transformation resolves the problem of an apparent proton migration of over 4 Å from the nucleophilic water to its LBHB position.

We finally address the problem of P_i_ release. Phosphate is tightly bound in three ways: 1) ionic ligation to magnesium, 2) an LBHB to O3B, estimated at 40–80 kJ mol^−1^,^15^ and 3) 8 H‐bonds from RhoGAP‐RhoA residues. These all need attenuation to promote phosphate dissociation. After trialing DFT structures, we computed shifting H^2^ to O1G (Figure 4 f). This has three features, meriting its description as an “exit” structure. The O1G−Mg bond length has increased 10 % to an abnormal 2.24 Å. The separation of O3G from O3B has increased to 2.99 Å, close to Van der Waals separation, replacing a bonding attraction of the LBHB by an anion–anion repulsion! Lastly, the Thr37 carbonyl group has moved to over 6 Å separation from PG, potentially initiating a Switch‐I change to OFF (Figure S4e, Supporting Information). It is thus possible that release of P_i_ is initiated by simple relocation of one mobile proton, synchronous with or following the movement of Switch‐I towards the OFF conformation and reinforced by dissociation of RhoGAP.

In summary, we have devised a general method for obtaining a crystalline intermediate complex for phosphoryl transfer of enzymes. The structural analysis of a novel RhoGAP‐RhoA‐GDP‐P_i_ complex shows how the GAP protein strongly stabilizes the bound phosphate intermediate with a LBHB linking the β‐ and γ‐phosphoryl groups. Two mobile protons are tracked from TS to intermediate complex and thence to release of phosphate, showing how sequential proton transfers complete the RhoGAP‐RhoA reaction mechanism for GTP hydrolysis (Figure S8, Supporting Information).

## Experimental Section

### General

Crystallographic methods and data are provided in the Supporting Information, as are details of the computational methods employed. Structural data for the RhoGAP‐RhoA‐GDP‐P_i_ intermediate complex and RhoA‐GDP product complex have been deposited in the Protein Data Bank as 6R3V and 5C4M.

## Conflict of interest

The authors declare no conflict of interest.

## Supporting information

As a service to our authors and readers, this journal provides supporting information supplied by the authors. Such materials are peer reviewed and may be re‐organized for online delivery, but are not copy‐edited or typeset. Technical support issues arising from supporting information (other than missing files) should be addressed to the authors.

Supplementary
