# Supplementary material for: A GAP‐GTPase‐GDP‐Pi Intermediate Crystal Structure Analyzed by DFT Shows GTP Hydrolysis Involves Serial Proton Transfers
Source: Chemistry. 2019 May 27;25(36):8484–8. doi: 10.1002/chem.201901627 (PMC6771576; doi:10.1002/chem.201901627)
Supplement: Supplementary file 1 — Supplementary [file CHEM-25-8484-s001.pdf]

CHEMISTRY
A European Journal

Supporting Information

A GAP-GTPase-GDP-P_i Intermediate Crystal Structure Analyzed by DFT Shows GTP Hydrolysis Involves Serial Proton Transfers

Robert W. Molt, Jr.+,[b, c] Erika Pellegrini+,[d] and Yi Jin+*[a]

chem_201901627_sm_miscellaneous_information.pdf

A GAP-GTPase-GDP-Pi Intermediate Crystal Structure Analyzed by DFT Shows GTP Hydrolysis Involves Serial Proton Transfers

Dr. Robert W. Molt, Jr., † [b,c] Dr. Erika Pellegrini, †[d] and Dr. Yi Jin†*[a]

[a] Cardiff Catalysis Institute, School of Chemistry, Cardiff University, Cardiff, CF10 3AT, UK.
[b] Department of Biochemistry & Molecular Biology, Indiana University School of Medicine, Indianapolis, Indiana 46202, United States
[c] ENSCO, Inc., 4849 North Wickham Road, Melbourne, Florida 32940, United States
[d] European Molecular Biology Laboratory, 71 Avenue des Martyrs, CS 90181, 38042, Grenoble, Cedex 9, France
[*] To whom correspondence may be addressed. Email: Jiny6@cardiff.ac.uk
† These authors contributed equally to the work.

Table of Contents

Experimental and Methods 2

Gene Expression and protein purification for RhoA and RhoGAP. 2

Crystallization and Structure refinement. 2

Crystallization procedures for RhoGAP-RhoA-GDP-$H_2PO_4^-$ complex. 2

Diffraction data collection and refinement method for RhoGAP-RhoA-GDP-$H_2PO_4^-$. 3

Crystallization procedures for Rho-GDP complex. 3

Diffraction data collection and refinement method for RhoA-GDP. 3

NMR Measurements to detect Pi binding to the RhoGAP-RhoA-GDP. 4

Determining Hydrogen Positions via Quantum Mechanics Calculations. 4

Tables and Figures 7

Table S1. Data collection and refinement statistics. 7

Table S2. Computed GS structures showing Hydrogen bonding involving Pi in various loci for the two mobile protons. 8

Figure S1. Phosphate titration experiment monitored by ^{1}H,^{13}C-TROSY NMR. 13

Figure S2. The comparison between the electron density maps during model fitting. 14

Figure S3. New high resolution RhoA-GDP product complex. 15

Figure S4. Comparison of RhoA-GDP product complex with transition state (analog) structures. 16

Figure S5. 8 asymmetric units of RhoGAP-RhoA-GDP-Pi complex 17

Figure S6. The active site inner surface of the RhoGAP-RhoA-GDP-Pi intermediate complex. 18

Figure S7. The previously calculated TS leading to various protonation state on Pi. 19

Figure S8. The H-bonding network highlighted through the whole catalytic cycle, completed by the intermediate proposed. 21

References: 21

Experimental and Methods

Gene Expression and protein purification for RhoA and RhoGAP. Plasmids expressing N-terminal GST-tagged RhoGAP (fragment 198–439) and N-terminal GST-tagged GST-$RhoA_{F25N}$ were generously provided by Dr. K. Rittinger (MRC National Institute for Medical Research, London). Both proteins were expressed in *E. coli.* BL21-Rosetta-pLysS or Rosetta 2 strains in LB media. Expression was induced with 1 mM IPTG for 6 h at 30 °C or overnight at 20 °C. Both RhoA and RhoGAP were purified following the same protocol. After cells were lysed by sonication in Lysis Buffer (50 mM Tris, pH 7.6, 50 mM NaCl, 5 mM $MgCl_2$, 1 mM DTT), the clear lysate was loaded on glutathione agarose column and incubated at 4 °C on a rotating wheel for 1 h before being washed with thrombin digestion buffer (50 mM Tris, pH 8.0, 150 mM NaCl, 5 mM $MgCl_2$, 1 mM DTT). The on-column thrombin digestion was carried out on beads overnight at 4 °C on a rotating wheel and the flow-through containing impure RhoGAP or RhoA was collected. The protein was further purified on a pre-equilibrated S75 Superdex Gel filtration column (50 mM Tris, pH 8.0, 150 mM NaCl, 5 mM $MgCl_2$, 1 mM DTT). RhoA used for crystallization was additionally purified by size exclusion column in a buffer of 50 mM Bis-Tris (pH 6.0), 150 mM NaCl, 5 mM $MgCl_2$, and 1 mM DTT. The protein eluted as monomer with GDP bound that co-purified from the *E. coli* cells. Protein was then concentrated and immediately set up for crystallization experiments.

Crystallization and Structure refinement.

Crystallization procedures for RhoGAP-RhoA-GDP-$H_2PO_4^-$ complex. The initial crystals of RhoGAP-RhoA-GDP-AlF_4^- were obtained using the crystallization conditions that are similar to the ones used for pdb **1tx4**: 20 % PEG2000 MME (Sigma), 110 mM $(NH_4)_2SO_4$, 100 mM MES, pH 6.0, 10 mM $MgCl_2$, 3 mM DTT, 3 mM NaN_3, 20 mM Tris-HCl pH 7.4, with 1 mM $AlCl_3$ and 10 mM NH_4F. Crystals were then transferred to a drop of the washing buffer containing 20% PEG2000 MME, 110 mM $(NH_4)_2SO_4$, 100 mM MES, pH 6.0, 3 mM DTT, 3 mM NaN_3, 20 mM Tris-HCl, pH 7.4, and 3 mM deferoxamine (DFO) for 2 h. This step was repeated, as necessary for complete depletion of aluminum and minimization of magnesium fluoride binding in the crystal. At this stage, selected crystals were flash-cooled with liquid nitrogen for data collection after being dipped into cryoprotectant (20% PEG2000 MME, 110 mM $(NH_4)_2SO_4$, 100 mM MES and 20% glycerol). Diffraction data latterly showed a RhoGAP-RhoA-GDP-MgF_3^- complex formed in the crystal (Figure S2a). The remaining

crystals were then transferred into a drop of the washing buffer with supplement of 10 mM $MgCl_2$, and 200 mM $NH_4H_2PO_4$ at a final pH of 5.5 for further soak for 1 h. This step is required to form RhoGAP-RhoA-GDP-$H_2PO_4^-$ complex. The same cryoprotectant was used (20% PEG2000 MME, 110 mM $(NH_4)_2SO_4$, 100 mM MES, and 20% glycerol) before these crystals were flash-cooled with liquid nitrogen.

Diffraction data collection and refinement method for RhoGAP-RhoA-GDP-$H_2PO_4^-$. Diffraction data were collected at 100 K with wave length 0.91731 Å, integrated with XDSGUI[1] and scaled with Aimless in CCP4i.[2] The RhoGAP-RhoA-GDP-$H_2PO_4^-$ structure (PDB **6r3v**) was solved by molecular replacement with MOLREP[3] in Collaborative Computational Project Number 4 suite (CCP4i)[2] using the coordinates of RhoGAP-RhoA-GDP-AlF_4^- (PDB **1tx4**) as the search model and refined by REFMAC5[4] using the maximum likelihood method. The model building was interactively done by using σA-weighted electron density maps with coefficients $2F_o$-F_c and F_o-F_c in COOT.[5] To confirm the electron density is that for a tetrahedral phosphate, the electron density of the phosphate (PO4) was explored by alternative refinement using PDB ligands "MGF" and "HOH" (trigonal planar MgF_3 with an in-line H_2O) as the model with the maximum Mg-F bond length strain (1.95 Å). This showed a region of positive difference density in the F_o-F_c map over the Mg atom (Figure S2b) with a much smaller B factor (32.6 for Mg versus 40.4, 36.3, 36.3 for the three fluorines). When the bond length strain on the MGF was set to minimum, after 10 further rounds of refinement, the MGF became distorted to tetrahedral geometry and all three 'Mg-F' bonds were refined as being shorter than 1.6 Å (Figure S2c).

The RhoGAP-RhoA-GDP-MgF_3^- structure from the DFO depletion procedure has a resolution of 1.87 Å and was refined in the same way, but only for the purpose of comparison without deposition to Protein Data Bank to avoid duplication of **1ow3** (Figure S2a).

Crystallization procedures for Rho-GDP complex. RhoA-GDP crystals were obtained at 293 K by the sitting-drop method from solutions consisting of 10 mg mL^{-1} RhoA, 50 mM MES, pH 5.5, 150 mM NaCl, 5 mM $MgCl_2$, 1 mM DTT, 1 mM $AlCl_3$, 10 mM NaF, 10 mM GDP equilibrated against 24% (w/v) PEG3350 and 0.3 M NaCl. Plate crystals appeared overnight and were mounted directly from the mother liquor using a mesh loop and cryo-cooling as described previously.[6]

Diffraction data collection and refinement method for RhoA-GDP. Diffraction data were collected from cryocooled crystals at 1.3 Å on a PILATUS 6M detector on beamline ID29 at the European Synchrotron Radiation Facility, Grenoble, France. Data were processed with

XDS [1] and programs from the CCP4. The previously published structure of RhoA-GDP (PDB **1ftn**),[7] without removal of bound ligand and water, was used as a search model for MOLREP.[3] Refinement was carried out alternately with REFMAC5[4] and by manual rebuilding with the program COOT.[5] Ligands were included in the final rounds of refinement. Model validation was performed using MolProbity.[8] Data collection and refinement statistics are summarized in Table S1. Figures were produced with the PyMOL Molecular Graphics System, Version 1.5.0.4 Schrödinger, LLC.[9]. Hydrogen bond distances were also measured using PyMOL and all those reported below have X-H-Y angles >145° (Figure S3 and S4).

NMR Measurements to detect Pi binding to the RhoGAP-RhoA-GDP. 2D ^{1}H, ^{13}C-TROSY experiments for phosphate titration were recorded on a Bruker Avance 800 MHz spectrometer equipped with a 5 mm four-channel cryoprobe. All spectra were recorded at 25 °C.

To assess the viability of determining the structure of a RhoGAP-RhoA-GDP-Pi intermediate complex, 2 M sodium phosphate stock in 100% D_2O (pD 6.0) was titrated to a maximum concentration of 1.0 M into a 0.5 mM ^{13}C,^{15}N-labeled RhoA-GDP (GDP bound 1:1) with 0.5 mM GAP in 150 mM NaCl, 10 mM $MgCl_2$ in 100 % D_2O at pD 6.0. Changes in the ^{13}C-TROSY for RhoGAP-RhoA-GDP complex were monitored as diagnostic of the binding of Pi. However, the sole changes observed in chemical shift were from residues on the surface caused by the 'salting-out' effect rather than by a Pi binding event in the active site. This indicates that for both complexes, the dissociation constant of Pi is substantially greater than 1.0 M (Figure S1). Hence there is no realistic possibility of obtaining a structure of a RhoGAP-RhoA-GDP-Pi intermediate complex co-crystallized from solution.

Determining Hydrogen Positions via Quantum Mechanics Calculations. We took the RhoGAP-RhoA-GDP-Pi crystal structure and selected an active site region (Figure 3) chosen to have all the key H-bonds that stabilize the GDP+Pi system and the appropriate ligands for the catalytic magnesium. In particular, this 210-atom model includes components of residues 12-20, 36-38, 59-63 (of chain B), Arg85' (of chain A), the catalytic magnesium and its two water ligands, methyl pyrophosphate, and $H_2PO_4^-$. We also included WAT698 as it serves to stabilize GDP. Non-active methyl groups were removed and carbons at which the model would extend beyond the QM zone were converted into methyl groups (Figure 3). Given that the purpose is to study the "microsolvated" region around the phosphates, residues far away from the region of interest were truncated (the electronic environment will be stable). In the same fashion, the guanosine in the GTP is truncated to a methyl group. All locked branch points that represent methyl groups approximating the rest of the protein environment are depicted $-CH_3$

in Figure 3. This procedure has been previously used to describe TSA crystal structures of RhoA-RhoGAP with KS-DFT to within the uncertainty of the experimental measurement.[10] Our models for the ground state (GS) of the GDP-Pi intermediate complexes were obtained using Kohn-Sham Density Functional Theory (KS-DFT). We used the M06-2X functional formulation of KS-DFT.[11] A cc-pVDZ basis set was used to represent single-particle wave-functions[12] for carbon, hydrogen, oxygen, and nitrogen atoms, while the cc-pVTZ basis was used for magnesium and phosphorus atoms.[12] Initially, the computation utilized cc-pVDZ for all atoms and an integration grid consisting of 99 radial points and 590 solid-angle points in the Lebedev grid, applying standard optimization algorithms to find the ground state structure. We added basis functions in the manner most conducive of better representing the active site and improved the phosphorus basis set to cc-pVTZ to represent its polarizable electron density with more accuracy. We also increased the magnesium basis set given its strong electron polarization. All oxygen atoms in the phosphate moieties used aug-cc-pVDZ, apart from inorganic phosphate, and O3B, which had aug-cc-pVTZ because of their importance.[13] The structure was considered optimized when the force on all nuclei fell below 1 μHartree/Bohr. The SCF was considered converged when the density matrix residual was less than 10^{-6}. We optimized the geometry of the resulting active site model (210 total atoms) to obtain the GS using standard algorithms,[14] as implemented in the Gaussian09 software package.[15]

We considered 7 possible hydrogen atom placements. The simplest case considered is the "immediate proton migration" of one of the two protons to either O1G, O2G, or O3G. By immediate migration, we mean the rotamer of the proton following an immediate migration from the nucleophilic water to the relevant oxygen.[10] We refer to these rotamers as being the "thermodynamic protonation states," because these rotamers presumably are more thermodynamically favorable in that they are stabilizing the leaving group. In each of these 7 possible protonation states, we selected a viable initial hydrogen position, and proceeded with the optimization.

Having optimized these 7 structures, we then compared the position of the heavy atoms in the active site region to the crystal structure. In each case, we removed D59 from the comparison; as it is on the boundary of the simulation, it is more prone to vacuum boundary artefacts, and also for WAT698. We used this methodology to compute seven GS structures with the two 'mobile' hydrogens (from the initial water molecule) apportioned variously on oxygens of the PG phosphate. Six of these have data for the H-bonds involving the four PG oxygens and O3B

listed in Table S2 Entries 2-7 and five were built into a viable progression of proton migrations shown in Figures S7b-S7f.

Tables and Figures

Table S1. Data collection and refinement statistics.

	RhoGAP-RhoA-GDP-Pi complex	RhoA-GDP
**PDB**	**6r3v**	**5c4m**
**Data collection**		
Space group	C2 2 2_1	$P2_12_12_1$
Cell dimensions		
a, *b*, *c* (Å)	114.1, 122.8, 67.2	31.80, 66.27, 83.68
α, β, γ (°)	90.0, 90.0, 90.0	
Resolution (Å)	43.5 - 1.75 (1.78 -1.75)	35.38 (1.37-1.3)
R_{sym} or R_{merge}	0.043 (1.307)	0.037 (0.265)
$I / \sigma I$	19.1 (1.4)	20.3 (5.1)
Completeness (%)	100.0 (99.9)	99.5 (99.3)
Redundancy	6.7 (6.7)	4.6 (4.5)
**Refinement**		
Resolution (Å)	83.6 - 1.75	20.3 - 1.3
No. reflections	45459	44107
R_{work} / R_{free}	0.1785 / 0.2068	0.1556/0.1843
No. atoms		
Protein	2927	1405
Ligand/ion	59	29
Water	254	180
B-factors		
Protein	38.8	21.5
Ligand/ion	44.4	12.8
Water	41.2	31.9
R.m.s. deviations		
Bond lengths (Å)	0.016	0.013
Bond angles (°)	1.76	1.636

Table S2. Computed GS structures showing Hydrogen bonding involving Pi in various loci for the two mobile protons.

Entry	Bond	D-H /Å	H---A / Å	D----A / Å	Angle D-H-A /°	Comment	Q_{DA}[a]
**1. O4→T37 & O4→Q63 " TS structure**	K18–O2G	1.05	1.64	2.67	164.25	**NB** Oxygens named to identify with the equivalent atoms in the GDP+Pi structures	100.15
	O4G-Q63	0.99	1.71	2.69	174.34		101.95
	O4G-T37	0.99	1.75	2.72	168.84		96.48
	T37(NH)-O1G	1.03	1.76	2.78	169.27		96.18
	R85'–O3G	1.03	1.77	2.77	162.99		92.08
	R85'-O3B	1.03	1.81	2.78	155.15		85.72
"	Q63(NH)–O3G	1.04	1.84	2.88	175.33		95.29
"	G62-O2G	1.02	1.84	2.84	163.47		99.68
"	***A15–O3B***	*1.03*	*1.81*	*2.74*	*148.34*	Poor angle and Q_{DA} is 2 s.d. from mean so omit from discussion	*81.96*
"	**Mg-O1G**			**2.09**		**TOTAL Q_{DA} score**	**849.5**
						**Mean Q_{DA} score**	**94.39**
						**Mean (Omit A15-O3B)**	**95.94 ± 5.18**
**2. O4→T37 & O3→Q63**	O4G–T37	0.99	1.70	2.61	150.70		88.65

“	G62-O2G	1.03	1.73	2.74	163.77		94.65
“	R85’–O3B	1.06	1.77	2.61	133.2		75.25
“	K18–O2G	1.04	1.78	2.77	154.84		86.99
“	T37(NH)-O1G	1.02	1.79	2.77	158.53		88.56
“	O3G-Q63(C=O)	0.99	1.90	2.86	167.87		88.35
“	R85’–O3G	1.02	2.00	3.02	170.82		85.41
“	*Q63(NH)–O3G*	*1.02*	*2.22*	*3.02*	*138.48*	Poor angle and Q_{DA} is >3 s.d. from mean so omit from discussion	*62.38*
	A15–O3B	1.04	1.66	2.65	157.44	Gives significant stabilization.	94.94
“	**Mg-O1G**			**2.24**		**Total for 7 bonds** (omit Q63-O3G)	**702.8**
“	**O3G-O3B**			**2.98**		**Mean Q_{DA} score** (omit Q63-O3G)	**87.9 ± 6.1**
**3. O4→T37 & O3G→O3B**	O3G–O3B	1.04	1.42	2.45	168.01	Good LBHB	118.32
“	R85’–O3B	1.05	1.63	2.66	168.49		103.35
“	O4G–T37	0.99	1.71	2.70	173.25		101.32
“	T37(NH)-O1G	1.02	1.74	2.73	161.52		92.83
“	G62-O2G	1.03	1.75	2.70	151.77		86.73
“	K18–O2G	1.04	1.78	2.78	159.50		89.61
“	R85’–O3G	1.03	1.93	2.92	163.03		84.47
“	Q63–O3G	1.02	1.97	2.98	169.75		86.17
“	*A15–O3B*	*1.02*	*1.89*	*2.74*	*139.0*	Poor angle and Q_{DA} is ~2 s.d. from mean so omit from discussion	*73.55*

"	**Mg-O1G**			**2.00**		**Total for 8 bonds** (Omit Q63-O3G)	**762.8**
"						**Mean Q_{DA} score** (Omit Q63-O3G)	**95.3 ± 11.6**
**4. O4→Q63 & O3G→O3B**	O3G–O3B	1.06	1.38	2.43	169.75	Best LBHB	123.01
"	G62-O2G	1.00	1.65	2.65	163.32		98.98
"	O4G–Q63	1.00	1.65	2.65	174.55		105.79
"	K18–O2G	1.04	1.70	2.72	163.01		98.79
"	T37(NH)-O1G	1.02	1.82	2.81	162.57		89.32
"	*R85'–O3B*	*1.05*	*1.86*	*2.65*	*128.98*	*Very poor angle*	*69.34*
"	Q63–O3G	1.02	1.94	2.96	176.32		90.89
"	R85'–O3G	1.03	1.95	2.95	163.49		83.84
"	*A15–O3B*	*1.02*	*1.98*	*2.77*	*132.77*	Poor angle and Q_{DA} is >s.d. from mean so omit from discussion	*67.06*
"	**Mg-O1G**			**2.05**		**TOTAL Q_{DA} score** (Omit R85'-O3G)	**690.62**
						**Mean**	**95.0 ± 15.87**
						**MEAN** (Omit R85'-O3G & A15-O3B)	**98.7 ± 13.0**
**5. O4→Q63 & O3B→O3G**	O3B–O3G	1.02	1.55	2.57	173.40	Not quite LBHB	111.87
"	K18–O2G	1.06	1.61	2.65	162.65		101.02
"	O4G–Q63	0.99	1.72	2.71	173.73		101.01
"	G62-O2G	1.03	1.75	2.74	161.45		92.26

"	R85'–O3G	1.04	1.76	2.77	166.46		94.58
"	T37(NH)-O1G	1.02	1.81	2.82	166.44		91.96
"	Q63–O3G	1.03	1.92	2.94	176.31		91.83
"	*R85'–O1G*	*1.02*	*2.34*	*3.33*	*162.99*	Long bond and Q_{DA} is >2 s.d. from mean so omit from discussion	*69.66*
"	**Mg-O1G**			**2.05**			
						**TOTAL Q_{DA} score** for 7 H-Bonds	**684.5**
						**Mean Q_{DA} score**	**97.8 ± 7.4**
**6. O4G→Q63 & O1G "**	**R85'–O3B**	1.06	1.56	2.92	166.42		106.68
	**G62-O2G**	1.03	1.69	2.60	161.66		95.66
"	**R85'–O3G**	1.03	1.74	2.77	170.76		98.14
"	**K18–O2G**	1.04	1.77	2.78	160.72		90.80
"	**Q63(NH)–O3G**	1.03	1.78	2.80	172.43		96.9
"	**O4G-Q63(C=O)**	0.99	1.78	2.61	170.68		104.37
	**T37(NH)-O4G**	1.02	2.18	3.06	143.57		95.89
"	**Mg-O1G**			**2.24**		*Only 6 H-bonds and no acceptor for the O1G Donor.*	
"						**Total Q_{DA} score**	**688.84**
						**Mean**	**98.35 ± 5.45**

**7. O4G→T37 & O1G**	**G62-O2G**						
	R85'–O3B	1.06	1.58	2.61	163.41		103.4
	O4G-T37(C=O)	0.98	1.78	2.68	150.92		84.8
	R85'–O3G	1.03	1.84	2.78	150.0		81.5
	K18–O2G	1.04	1.90	2.94	173.4		91.26
	Q63(NH)–O3G	1.02	2.20	2.82	117.37		53.4
	T37(NH)-O3G	1.02	2.48	3.06	115.56	Very weak	46.6
	O1G-T60(C=O)	0.97	2.49	3.38	108.14		43.43
	**O1G-Mg**			**2.17**			
	**O3G—O3B**			**2.93**		**Total Q_{DA} score**	**504.4**
						**Mean**	**72.1 ± 23.9**

[a] Q_{DA} is the quotient for D-H—A bond angle divided by H—A bond length, as defined in the main text; units of degrees/Å.

All Oxygens named to identify with the equivalent atoms in the RhoGAP-RhoA-GDP-Pi structures.

Figure S1. Phosphate titration experiment monitored by ^{1}H,^{13}C-TROSY NMR. The ^{1}H and ^{13}C chemical shifts of the four peaks (indicated by arrows) that are typical for βH and βC of threonine within a protein are plotted against the various concentrations, showing phosphate does not bind below 1 M concentration to RhoGAP-RhoA-GDP under the condition of the experiment. The threonines are chosen because Thr19 and Thr37 are from Switch-I and Thr60 is from Switch-II. These residues would undergo bigger chemical shift change in the case of phosphate binding. The colors chosen in the 2D NMR are representing the following Pi concentrations for clarity: green 0.5 mM, black 50 mM, red 500 mM, blue 1000 mM.

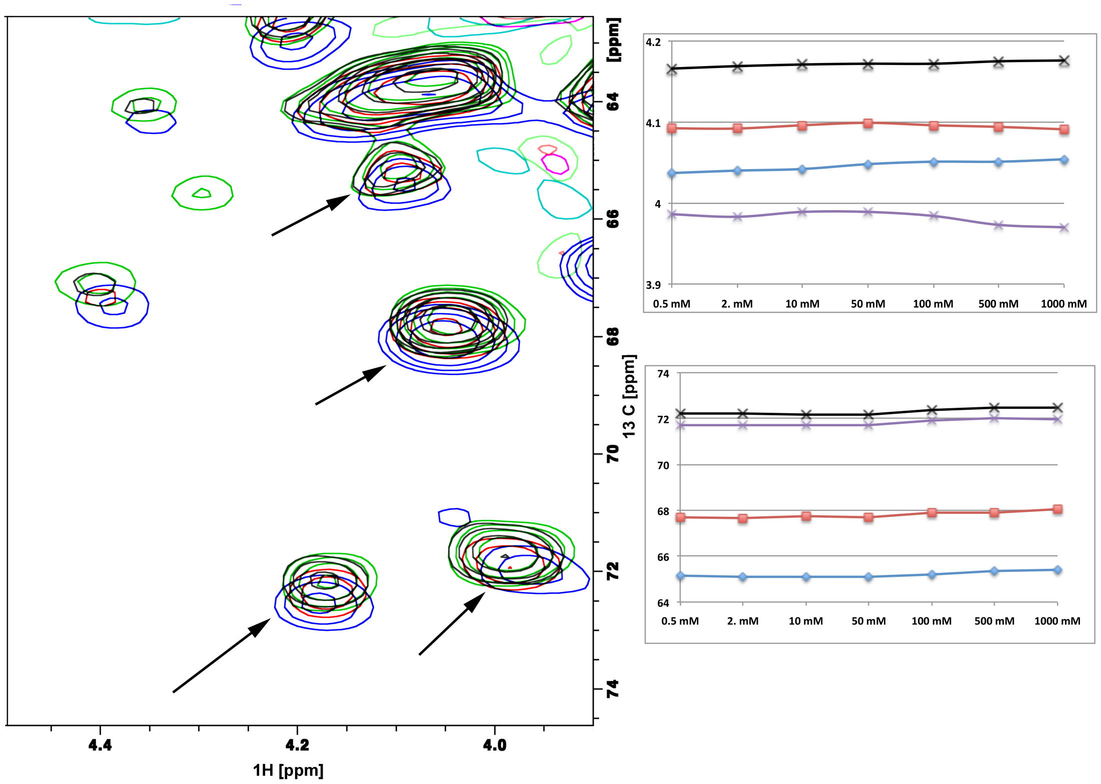

Figure S2. The comparison between the electron density maps during model fitting. a) In situ RhoGAP-RhoA-GDP-MgF_3^- TSA complex after the crystals being washed in a drop containing DFO but no $AlCl_3$, $MgCl_2$ and NH_4F. b) For the data set of the RhoGAP-RhoA-GDP-Pi complex, the F_o-F_c map shows a region of positive difference density (in green mesh) over the central Mg atom, when the density of the phosphate was refined using PDB ligands "MGF" and "HOH" as the model with the maximum Mg-F bond length strain. The geometry of the MGF is distorted out of the plane towards tetrahedral. c) The refined RhoGAP-RhoA-GDP-Pi intermediate complex. Electron density maps shown in grey mesh are the σ_A-weighted $2F_o - F_c$ omit map contoured at 1σ (0.24 electrons per Å^3). d) The comparison of B-factors after normalization. The resolutions of the structures are comparable with each other: the deposited MgF_3^- structure (**1ow3**, 1.80 Å), in-situ MgF_3^- structure (1.87 Å), GDP-Pi structure (**6r3v**, 1.75 Å). The missing density region in the GDP-Pi structure is highlighted in yellow dash in the cartoon.

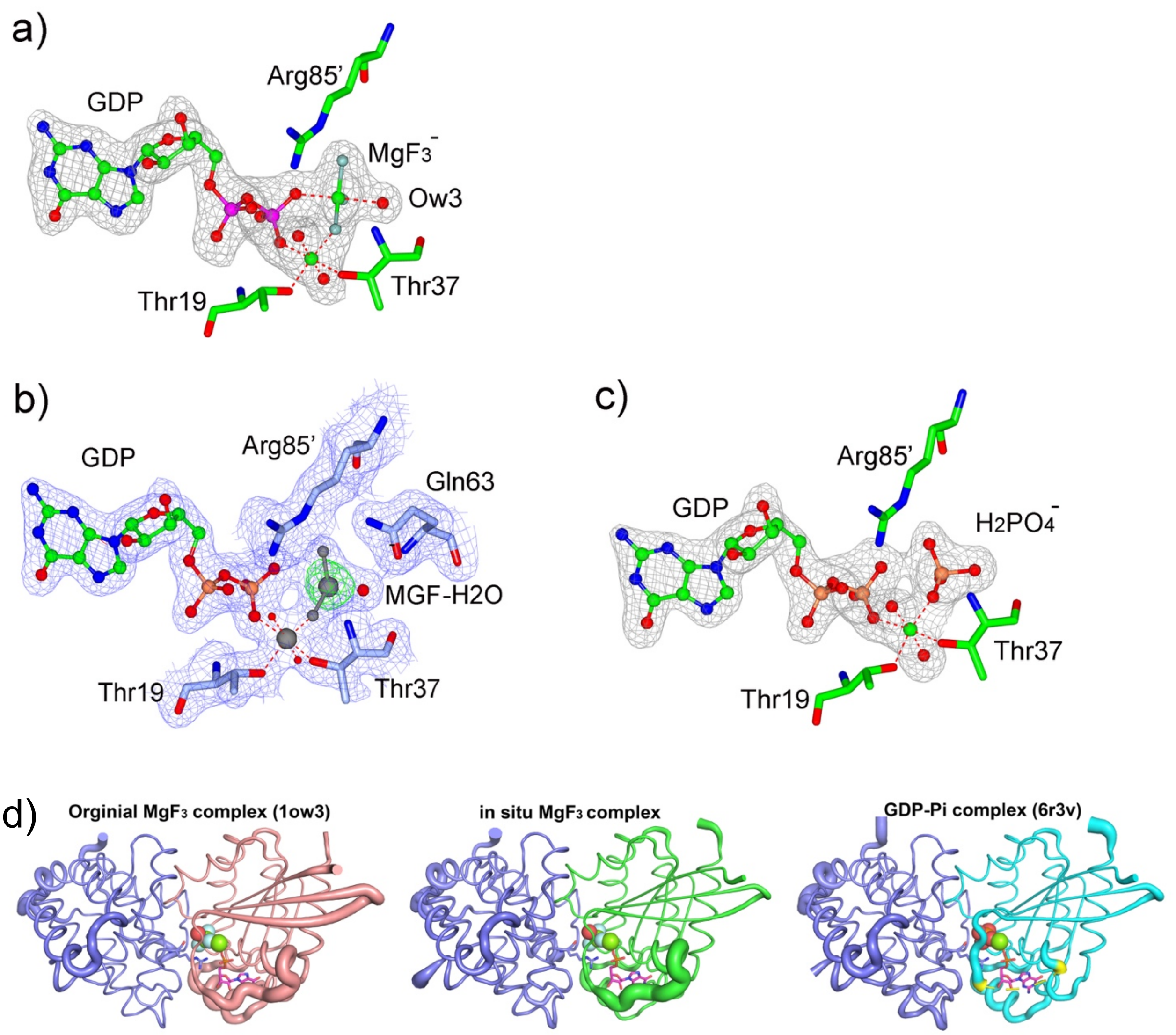

Figure S3. New high resolution RhoA-GDP product complex. a) The electron density (green mesh) of the key residues in Switch-II in the high-resolution structure of RhoA-GDP (**5c4m**). Electron density maps in green mesh are σ_A-weighted $2F_o - F_c$ contoured at 1σ. b) Overlay of RhoA-GDP product structures for the high resolution RhoA-GDP (**5c4m** in gray) and RhoA-GDP (**1ftn** in cyan). switch II (residues 61 - 78) is highlighted in lime in 5m4c, whereas the same region with the missing density in 1ftn is highlighted in blue. The difference between the Switch-II region position is evident.

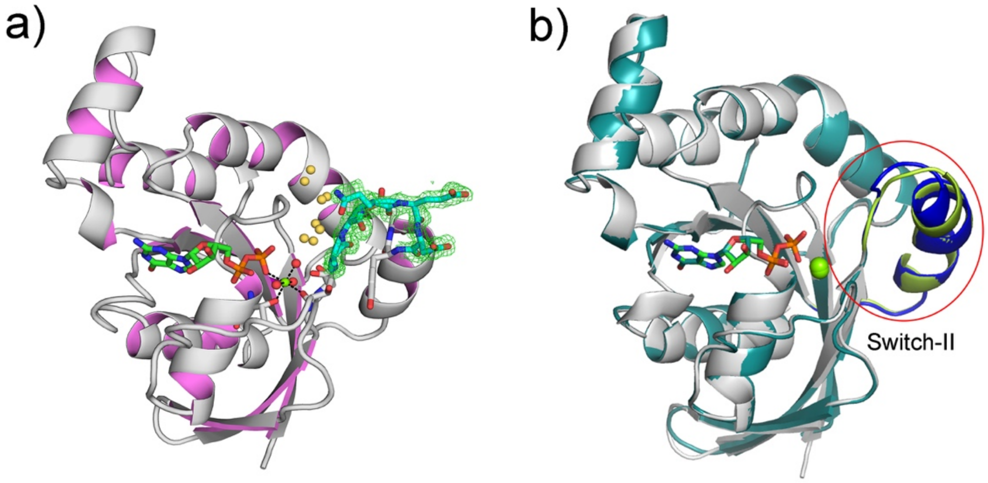

Figure S4. Comparison of RhoA-GDP product complex with transition state (analog) structures. a) The Switch-I (in magenta) and Switch-II (in cyan) in the structure RhoGAP-RhoA-GDP-MgF_3^- TSA complex are both in ON conformation. b) The Switch-I (in magenta) and Switch-II (in cyan) in the structure RhoA-GDP product complex are both in OFF conformation. c) The rotation of Thr37 is compared for the TS (purple sticks) and the product post-Pi release (yellow sticks). d) Scheme to show Thr37 coordinates the catalytic Mg^{II} with the sidechain hydroxyl group in the TS. e) Scheme to show Thr37 coordinates the catalytic Mg^{II} with the backbone carbonyl group after Pi leaves in the product state.

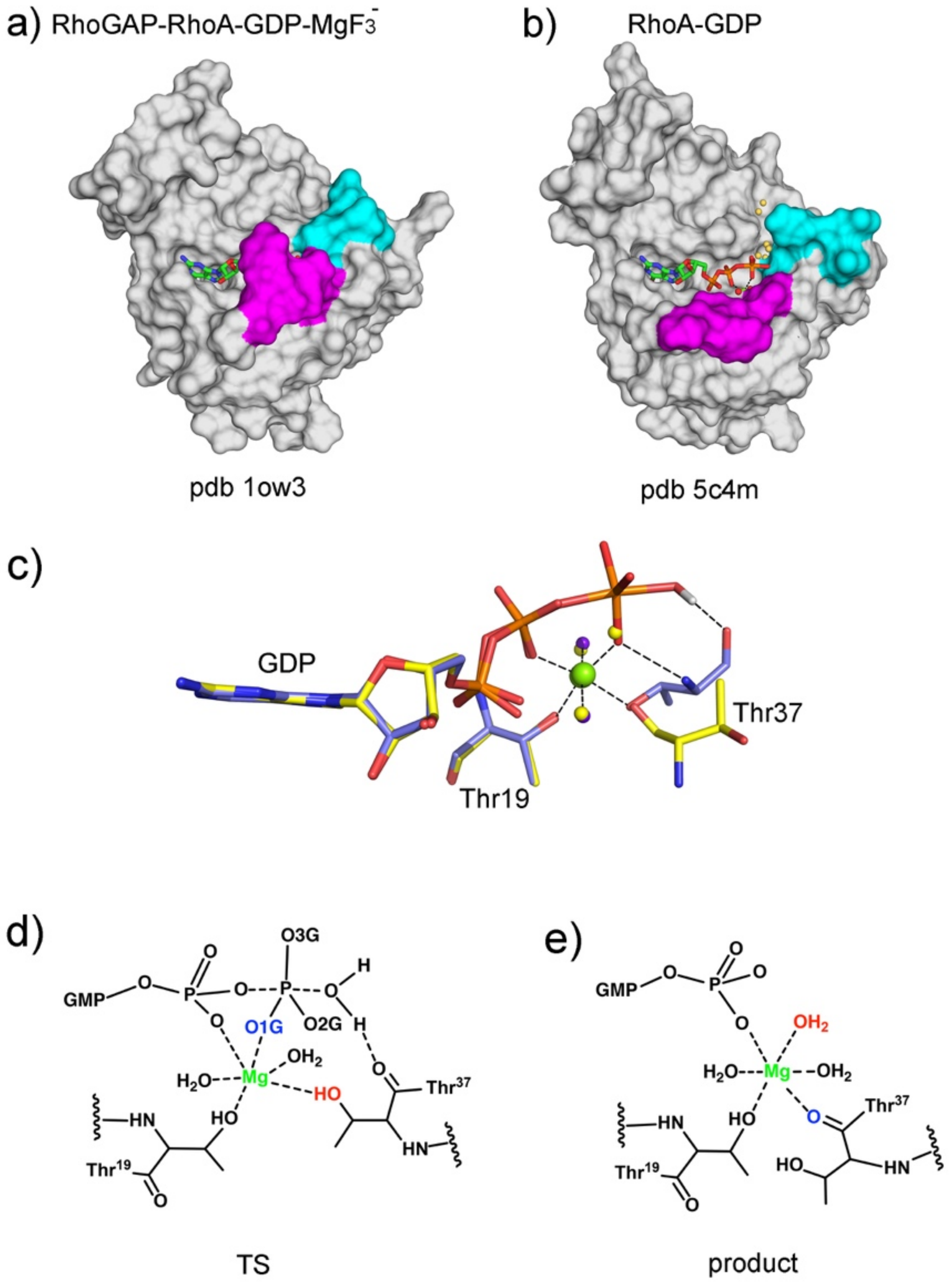

Figure S5. 8 asymmetric units of RhoGAP-RhoA-GDP-Pi complex consisted of RhoA (in gray) and GAP (in pink) in a unit cell of the crystal lattice in a Spacegroup $C222_1$. Switch-I (in green) and Switch-II (in yellow) are not on the surface of the proteins involved in crystal stacking.

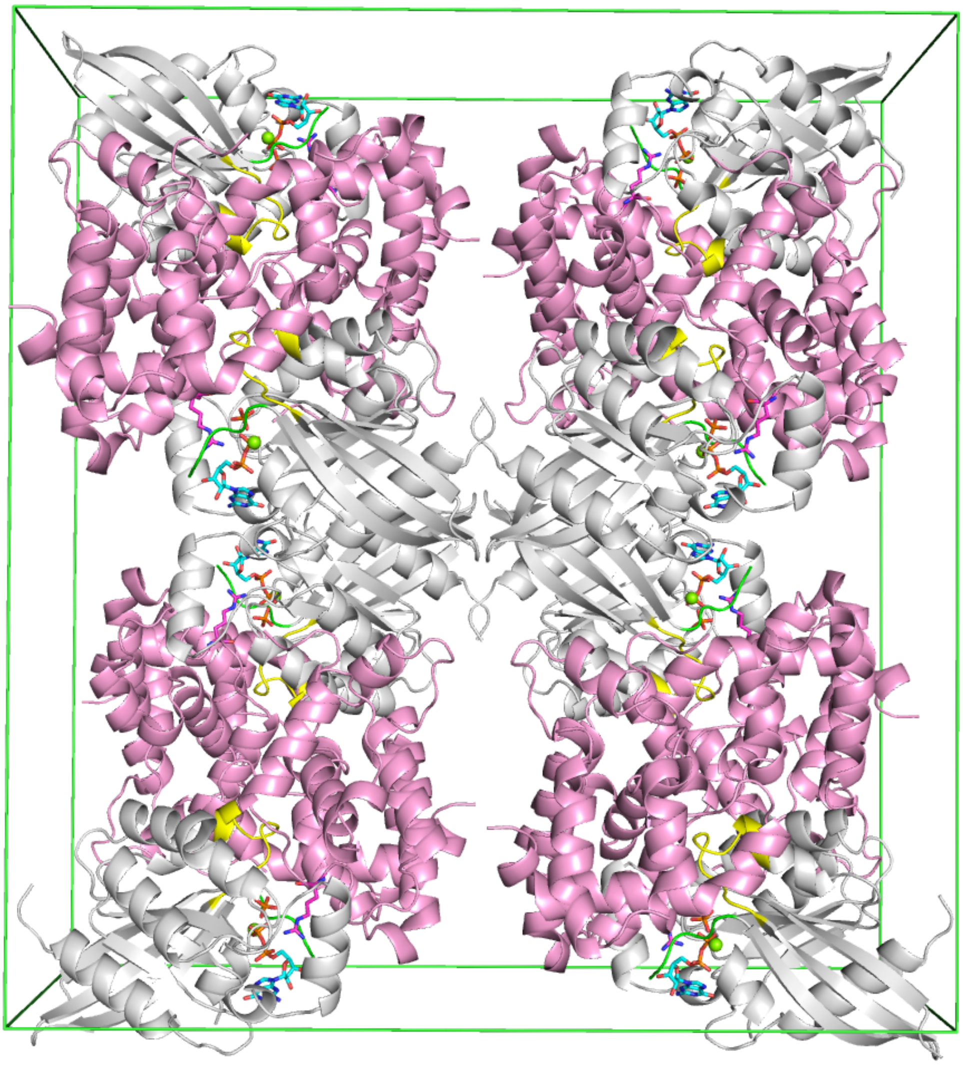

Figure S6. The active site inner surface of the RhoGAP-RhoA-GDP-Pi intermediate complex. It shows that, there is no space in the active site to accommodate an extra water molecule next to the Pi, even after the whole process of dissociation of AlF_4^- followed by binding of Pi during crystal soaking. The inner surface of the intermediate complex active site is in cyan. The residues from RhoA are in cyan sticks and RhoGAP in purple sticks. The GDP and Pi are in sticks. The key interactions are highlighted in black dashes; PA, PB and PG are in orange, nitrogen atoms are in blue, and oxygen atoms are in red.

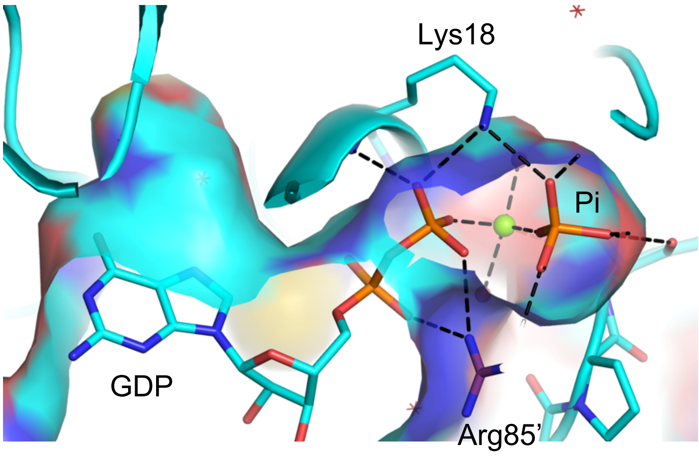

Figure S7. The previously calculated TS leading to various protonation state on Pi.

Figure S7a. TS. O4-T37 & O4-Q63 (Entry 1 Table S2). This is the computed transition state.[10] Both H^1 (gray) and H^2 (magenta) are located on O4G directed at Thr37(C=O) and Gln63(C=O) respectively. It shows good H-bonds between O3B and Arg85' and Gln63, and between O2B and Lys18 and Gly62. O3B accepts H-bonds from Ala15 and Arg85'. O3G and O3B are 2.6 Å apart. This DFT structure (red sticks) is introduced to initiate the mobile proton sequence that follows here. Rainbow coloring is used for the progression of migrations.

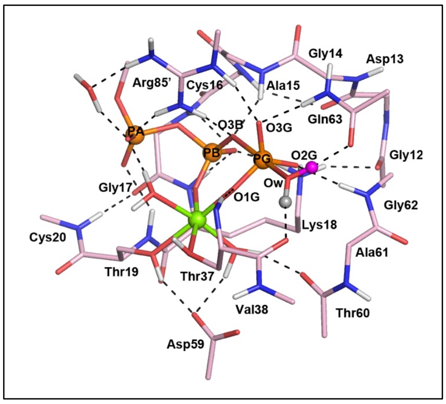

Figure S7b. O4-T37 & O3-Q63 (Entry 2, Table S2) has H^1 (gray) located on O4G directed at Thr37(C=O) and H^2 (magenta) located on O3G directed at Gln63(C=O) (orange sticks). It shows good H-Bonds between Pi and Arg85', Gln63, Gly62, Lys18, and Thr37. O3B accept H-bonds from Ala15 and Arg85'. This is designated the first DFT structure in the main text.

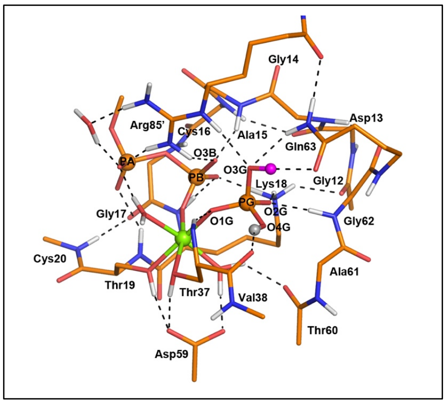

Figure S7c. O4-T37 & O3G-O3B (Entry 3, Table S2) has H^1 (gray) located on O4G directed at Thr37(C=O) and H^2 (magenta) located on O3G directed at O3B (yellow sticks). It shows good H-bonds between Pi and Arg85', Gln63, Gly62, Lys18, and Thr37. O3B accepts H-bonds from Ala15 and Arg85'. This is designated the second DFT structure in the main text.

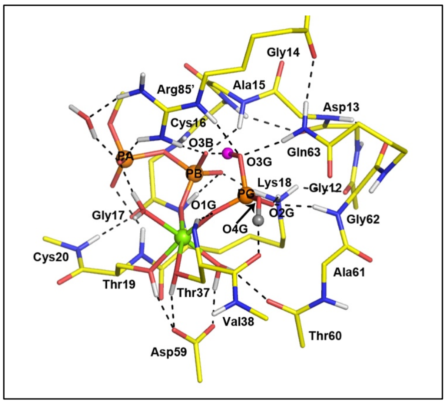

Figure S7d. O4-Q63 & O3G-O3B (Entry 4 Table S2) has H[1] (gray) located on O4G directed at Gln63(C=O) and H[2] (magenta) located on O3G directed at O3B (green sticks). It shows good H-bonds between Pi and Arg85', Gln63, Gly62, Lys18, and Thr37. O3B accept H-bonds from O3G, Ala15 and Arg85'. This is designated the third DFT structure in the main text.

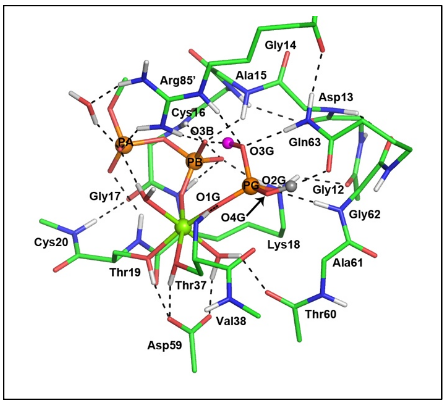

Figure S7e. O4-Q63 & O2B-O3B (Entry 5 Table S2) has H[1] (gray) located on O4G directed at Gln63(C=O) and H[2] (magenta) re-located on O3B directed at O3G (cyan sticks). It shows good H-bonds between Pi and Ala15, Arg85', Gln63, Gly62, and Lys18. But Arg85' has moved (N2 shifting 0.3 Å) and does not H-bond to O3B while Thr37 has moved 0.6 Å away from PG. O3B donates a H-bond to O3G and accepts one from Ala15. Thr37(C=O) is 4.50 Å from PG. This is designated the fourth DFT structure in the main text.

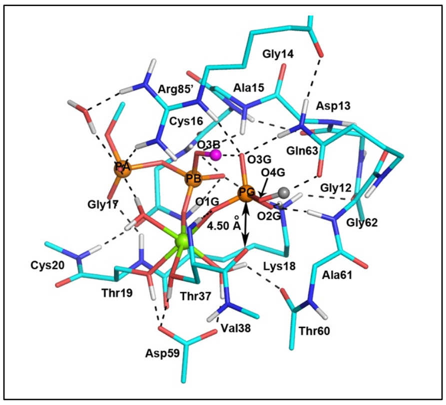

Figure S7f. O4G-Q63 & O1G (Entry 6 Table S2) has H[1] (gray) located on O4G directed at Gln63(C=O) while H[2] (magenta) located on O1G has no H-bond acceptor (indigo sticks). There are good H-bonds between Pi and Arg85', Gln63, Gly62, Lys18, and Thr37(N-H). O3B is 2.89 Å from O3G and accepts H-bonds from Ala15 and Arg85'. Thr37(C=O) is now over 6 Å from PG and has rotated about both phi- and psi-bonds. This is designated the final DFT structure in the main text.

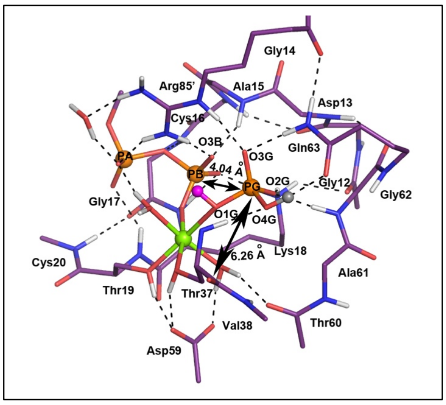

Figure S8. The H-bonding network highlighted through the whole catalytic cycle, completed by the intermediate proposed.

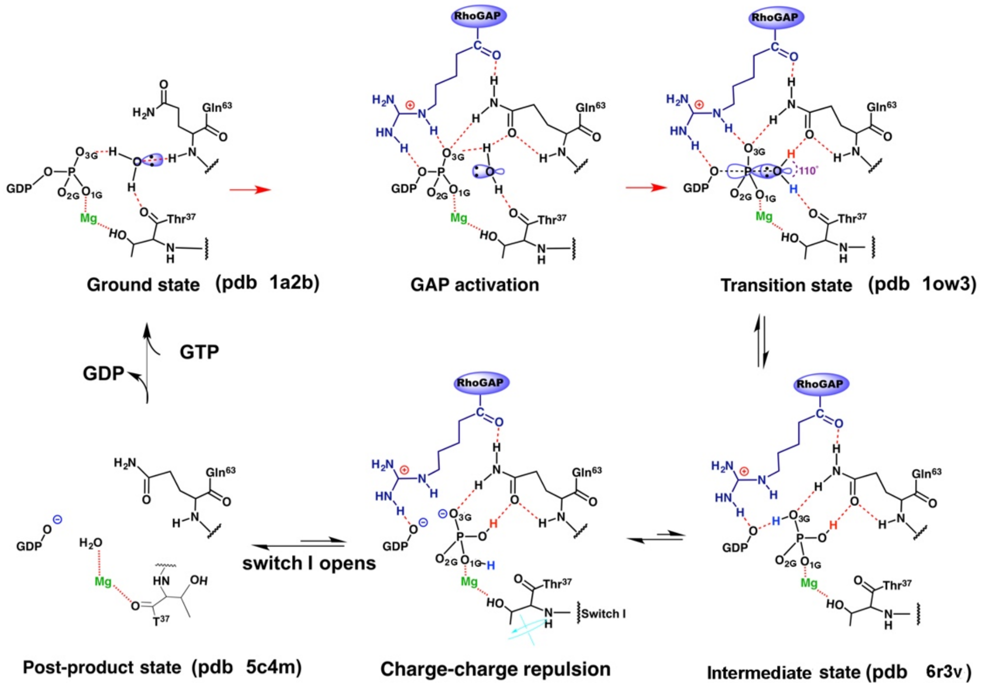

References:

[1] W. Kabsch, *Acta Crystallogr. D Biol. Crystallogr.* **2010**, *66*, 125-132.

[2] M. D. Winn, C. C. Ballard, K. D. Cowtan, E. J. Dodson, P. Emsley, P. R. Evans, R. M. Keegan, E. B. Krissinel, A. G. W. Leslie, A. McCoy, S. J. McNicholas, G. N. Murshudov, N. S. Pannu, E. A. Potterton, H. R. Powell, R. J. Read, A. Vagin, K. S. Wilson, *Acta Crystallogr. D Biol. Crystallogr.* **2011**, *67*, 235-242.

[3] A. Vagin, A. Teplyakov, *Acta Crystallogr. D Biol. Crystallogr.* **2010**, *66*, 22-25.

[4] G. N. Murshudov, A. A. Vagin, E. J. Dodson, *Acta Crystallogr. D Biol. Crystallogr.* **1997**, *53*, 240-255.

[5] P. Emsley, B. Lohkamp, W. G. Scott, K. Cowtan, *Acta Crystallogr. D Biol. Crystallogr.* **2010**, *66*, 486-501.

[6] E. Pellegrini, D. Piano, M. W. Bowler, *Acta Crystallogr. D Biol. Crystallogr.* **2011**, *67*, 902-906.

[7] Y. Wei, Y. Zhang, U. Derewenda, X. Liu, W. Minor, R. K. Nakamoto, A. V. Somlyo, A. P. Somlyo, Z. S. Derewenda, *Nat. Struct. Biol.* **1997**, *4*, 699-703.

[8] V. B. Chen, W. B. Arendall, III, J. J. Headd, D. A. Keedy, R. M. Immormino, G. J. Kapral, L. W. Murray, J. S. Richardson, D. C. Richardson, *Acta Crystallogr. D Biol. Crystallogr.* **2010**, *66*, 12-21.

[9] The PyMOL Molecular Graphics System, Version 2.0 Schrödinger, LLC.

[10] Y. Jin, R. W. Molt, J. P. Waltho, N. G. J. Richards, G. M. Blackburn, *Angew. Chem. Int. Ed.* **2016**, *55*, 3318-3322.

[11] a) Y. Zhao, D. Truhlar, *Theor. Chem. Acc.* **2008**, *120*, 215-241; b) N. Mardirossian, M. Head-Gordon, *J. Chem. Theor. Comput.* **2016**, *12*, 4303-4325.

[12] T. H. Dunning, *J. Chem. Phys.* **1989**, *90*, 1007-1023.

[13] R. A. Kendall, T. H. Dunning, R. J. Harrison, *J. Chem. Phys.* **1992**, *96*, 6796-6806.

[14] X. Li, M. J. Frisch, *J. Chem. Theor. Comput.* **2006**, *2*, 835-839.

[15] a) M. J. Frisch, G. W. Trucks, H. B. Schlegel, G. E. Scuseria, M. A. Robb, J. R. Cheeseman, G. Scalmani, V. Barone, B. Mennucci, G. A. Petersson, H. Nakatsuji, M. Caricato, X. Li, H. P. Hratchian, A. F. Izmaylov, J. Bloino, G. Zheng, J. L. Sonnenberg, M. Hada, M. Ehara, K. Toyota, R. Fukuda, J. Hasegawa, M. Ishida, T. Nakajima, Y. Honda, O. Kitao, H. Nakai, T. Vreven, J. A. Montgomery Jr., J. E. Peralta, F. Ogliaro, M. J. Bearpark, J. Heyd, E. N. Brothers, K. N. Kudin, V. N. Staroverov, R. Kobayashi, J. Normand, K. Raghavachari, A. P. Rendell, J. C. Burant, S. S. Iyengar, J. Tomasi, M. Cossi, N. Rega, N. J. Millam, M. Klene, J. E. Knox, J. B. Cross, V. Bakken, C. Adamo, J. Jaramillo, R. Gomperts, R. E. Stratmann, O. Yazyev, A. J. Austin, R. Cammi, C. Pomelli, J. W. Ochterski, R. L. Martin, K. Morokuma, V. G. Zakrzewski, G. A. Voth, P. Salvador, J. J. Dannenberg, S. Dapprich, A. D. Daniels, Ö. Farkas, J. B. Foresman, J. V. Ortiz, J. Cioslowski, D. J. Fox, Gaussian, Inc., Wallingford, CT, USA, **2009**; b) R. Dennington, Keith, T., and Millam, *GaussView, Version 5*, *Semichem Inc., Shawnee Mission, KS* **2009**.
